# Supplementary material for: Are carbon emissions trading and green financial instruments synergistic? -Comprehensive quantitative research based on content analysis
Source: PLoS One. 2024 Mar 7;19(3):e0298601. doi: 10.1371/journal.pone.0298601 (PMC10919601; doi:10.1371/journal.pone.0298601)
Supplement: S1 Data — (ZIP) [file pone.0298601.s001.zip › Data/地级市Z_F工作报告.zip!/╜¡╬≈/╕╙╓▌╩╨/└┤╫╘╣1⁄2╓┌║┼í╛┬φ┐╦╩2╛▌═°í┐_2019.docx]

各位代表：

　　现在，我代表市人民政府向大会报告工作，请予审议，并请各位政协委员和列席的同志提出意见。

　　一、2018年工作回顾

　　2018年，是全面贯彻党的十九大精神开局之年，是改革开放40周年。在省委、省政府和市委的坚强领导下，我们坚持以习近平新时代中国特色社会主义思想和党的十九大精神为指导，从更高层次贯彻落实习近平总书记对江西和赣州工作的重要要求，把中央打好三大攻坚战的部署要求贯穿经济社会发展全过程、各方面，全面贯彻省委“二十四字”【1】工作方针，坚持解放思想、内外兼修、北上南下，坚定不移打好我市六大攻坚战，纵深推进赣南苏区振兴发展，较好完成了市五届人大三次会议确定的目标任务。

　　——主要经济指标增幅稳居全省前列。实现生产总值2807.24亿元，增长9.3%；财政总收入459.51亿元，增长12.5%；固定资产投资增长11.3%；规模以上工业增加值增长9.5%；实际利用外资18.44亿美元，增长10.6%；城镇居民人均可支配收入32163元，增长8.8%；农村居民人均可支配收入10782元，增长11%；工业投资增长19.8%；民间投资增长18%；服务业增加值1272.7亿元，增长11.5%，这10项指标增幅全省第一。一般公共预算收入265.21亿元，增长8.1%；社会消费品零售总额901.71亿元，增长11.1%；出口总额287.44亿元，增长6.9%，增幅均稳居全省“第一方阵”。在经济下行压力加大的形势下，我市经济发展逆势上扬、领跑全省、极其不易！

　　——打好中央三大攻坚战成效明显。脱贫攻坚取得重大进展。瑞金以优异成绩脱贫摘帽，南康、寻乌、安远、石城、上犹、会昌基本具备脱贫摘帽条件，全年脱贫19.1万人、退出贫困村426个，贫困发生率降至2.45%。污染防治取得明显成效。中央环保督察年度整改任务全面完成，“回头看”反馈情况总体较好。废弃稀土矿山治理基本完成，近半个世纪的历史遗留问题有效破解。钨渣实现综合回收利用，制约钨产业发展的难题得到攻克。群众反映强烈的“三江六岸”【2】污染企业陆续启动搬迁，已拆除大烟囱5座。蓝天保卫战打了漂亮的“翻身仗”，中心城区PM2.5浓度均值优于省考核目标。水环境质量保持全省前列，5个断面劣Ⅴ类水全部消灭。19个省级以上工业集聚区污水处理设施全部建成联网，市生活垃圾焚烧发电厂等一批治根本的环保设施建成运行。45个已开工山水林田湖草生态保护修复项目完成投资94.3亿元，完成低质低效林改造113万亩，东江流域出境水质100%达标，河长制、湖长制、林长制【3】全面落实，南方重要生态屏障进一步筑牢。防范化解重大风险措施有力。全市总体债务率下降26.2个百分点，不良贷款率全省最低，房地产市场平稳健康发展，重点领域风险总体可控。中央三大攻坚战决策部署在赣南大地执行有力、精准高效、落地生根！

　　——我市六大攻坚战三年成果令人振奋。

　　“主攻工业、三年翻番”目标如期实现。规上工业企业主营业务收入、工业固定资产投资实现同口径翻番；规上工业企业从1188家增加到1890家，总量、增量均为全省第一；高新技术企业从137家增加到501家；“两城两谷一带”和各地首位产业加速形成壮大，家具产业集群产值突破1600亿元，新增省级重点工业产业集群5个、总数达11个，首位产业集中度从15%提高至45%；战略性新兴产业、高新技术产业增加值占规上工业比重达17.4%和33%，分别提高4.9和7.5个百分点；园区基础设施投资翻了两番、增幅连续三年全省第一；建成标准厂房1520万平方米、为三年计划的1.5倍。全市上下主攻工业氛围浓厚、思路清晰，形成了围绕主导产业和首位产业集群发展、创新发展、招大引强的良好势头。

　　新型城镇化实现历史性跨越。全市常住人口城镇化率突破50%。赣州成为全省唯一的省域副中心城市，省政府赋予44项省级管理权限、6个先行先试项目。成功创建全国文明城市、国家森林城市。文明大道、迎宾大道快速路建成通车，赣州进入“高架时代”！中心城区形成五区一体化发展新格局，建成区面积扩大至180平方公里、城市人口增加到179万人，昔日“小马拉大车”，如今“五马齐奔腾”。县城功能品位明显提升，龙南、大余、瑞金获得全国文明城市提名，崇义获评国家生态文明建设示范县和全国首批县级国家森林城市，上犹荣获全国美丽乡村创建先进县，大余丫山、全南南迳、宁都小布入选国家级特色小镇，南康家居小镇上榜全国最美特色小镇50强。全市城镇综合承载能力显著增强，城乡面貌焕然一新！

　　现代农业取得突破性进展。脐橙产业稳产稳收，柑橘黄龙病实现可控，赣南脐橙产区入选中国特色农产品优势区，品牌价值稳居全国榜首。蔬菜产业异军突起，三年新增钢架大棚12.16万亩，建成规模蔬菜基地20.4万亩，开行中欧蔬菜班列，赣南蔬菜正走向全国、走出国门，成为继脐橙之后又一农业富民产业。油茶产业持续壮大，三年新造高产油茶林37.5万亩，改造低产油茶林38.6万亩，赣南茶油获批国家地理标志证明商标，品牌价值跻身全国百强。成功创建2个国家级、28个省级现代农业示范园，信丰现代农业产业园被认定为国家级、系全省唯一。三年新增规上农产品加工企业58家，年加工20万吨脐橙的农夫山泉建成投产。三年新增国家地理标志证明商标12件、总量达25件，均列全省第一。我市现代农业发展找准了路径，设施农业、科技农业、规模农业迅速扩张，农业发展理念发生根本性转变！

　　（下转2版）　　（上接1版）

　　现代服务业蓬勃兴起。规上服务业企业数量实现翻番，服务业增加值突破千亿元，增速连续三年全省第一，占GDP比重达45.3%，超二产2.7个百分点，服务业支撑作用凸显。旅游接待人次突破1亿、总收入突破1000亿元。金融机构存、贷款余额分别超过5000亿元和4000亿元，存贷比达80%，实现历史性突破。国家标准A级物流企业总量、增量全省第一。赣州国际陆港从无到有、实现爆发式增长，获批肉类、汽车整车进口指定口岸，开行至盐田港、厦门港、广州港“同港同价”双向班列，开通中欧（亚）班列线路19条，年吞吐量突破40万标箱，成为全省开放提升的“引爆点”。

　　基础设施更加完备。通过三年努力，动车运营实现“零突破”，昌赣高铁全线铺轨，高铁时代即将到来！赣深高铁、兴泉铁路加快建设，长赣高铁、瑞梅铁路前期工作取得积极进展，“米”字形铁路网布局成型；广吉高速宁都段、宁定高速、兴赣高速建成通车，新增高速公路通车里程375公里，大广高速南康至龙南段扩容工程即将开工，“三纵三横六联”【4】高速公路网基本形成；黄金机场改扩建主体工程基本完工、旅客吞吐量及增速居全省支线机场之首，瑞金机场完成立项报批，全国性综合交通枢纽地位日益凸显。华能瑞金电厂启动二期两台百万千瓦机组主厂房建设，一期两台66万千瓦机组、二期两台百万千瓦机组的信丰电厂即将开工，赣州能源自我保障能力将全面形成！

　　三年来，全市累计实施六大攻坚战项目5462个、竣工3772个，建成了一批打基础、利长远、增后劲、惠民生的重大项目，破解了一批长期想解决而没有解决的难题，办成了许多过去想办而没有办成的大事实事，赣州进入了投资力度最大、干事创业氛围最浓、综合实力提升最快、城乡面貌变化最大、群众获得感最强的黄金发展期。实践证明，六大攻坚战是牵引赣南苏区振兴发展的有效路径和有力抓手，已成为全市上下的高度共识和自觉行动，在赣州深入人心！

　　在六大攻坚战的强力带动下，过去一年，各项工作开创了新局面：

　　一是产业发展迈上新台阶。“两城两谷一带”加速壮大。“新能源汽车科技城”引进的国机、凯马整车生产线建成试产，孚能科技经科技部认定为全省唯一“独角兽”企业【5】；“现代家居城”规上企业突破400家，“南康家具”成为全国首个以县级行政区划命名的工业集体商标、品牌价值蝉联全省制造业第一；“中国稀金谷”入驻“国字号”创新平台4个，新引进中科拓又达等项目23个；“青峰药谷”建成全省首个国家高层次人才产业园，青峰药业获批国家企业技术中心、1项科研成果获国家科技进步奖二等奖；“赣粤电子信息产业带”新引进10亿元以上项目22个，投资5亿美元的技研新阳等一批项目建成投产。园区平台加快升级，章贡高新区入选国家级绿色园区，信丰工业园被认定为省级高新区，兴国获批设立省级军民融合产业基地。现代农业提质增效。耕地保有量和基本农田保护目标全面实现，建成高标准农田33.27万亩。农业特色产业加快发展，黄鸡、麻鸭、灰鹅等家禽出栏1.2亿羽，创建生猪养殖标准化示范场111家，“赣南高山茶”集体商标已申请注册，上犹获评中国茶叶百强县。新增规模以上农业企业38家、“三品一标”【6】农产品65个，农产品质量安全合格率稳定在98%以上。现代服务业亮点纷呈。发起并成功举办首届“红军长征论坛”，章贡区极地海洋世界、会昌汉仙盐浴温泉、龙南南武当山景区、安远东生围景区、全南雅溪古村等项目建成运营，郁孤台历史文化街区获评“中国商旅文产业发展示范街区”，石城被认定为全省首批全域旅游示范区，大余获评全国森林旅游示范县。金力永磁A股上市，瑞京金融资产管理公司获批开业，金融控股集团成功组建，设立赣州发展基金、全省首支企业纾困发展基金，全国首家并购基金园【7】、赣南苏区企业路演中心【8】成立运营。投资20亿元的赣州综合物流园一期建成投运，赣州冷链物流中心主体工程完工，传化南北公路港获评全国优秀物流园区，顺丰科技首个无人机物流项目在南康试点并获全国首张运营牌照，智慧物流“吉集号”荣登中国产业互联网百强榜。南康、龙南、定南入选中国电商示范百佳县，寻乌、安远、石城进入全国贫困县农产品电商50强，瑞金获评全国返乡创业电商合作示范县，全市电子商务产业发展驶入快车道。

　　二是改革开放实现新突破。“放管服”改革深入推进。在全省率先开展企业开办、工程项目审批、不动产登记三个“一窗办”改革，率先提供错时延时服务，“多证合一”改革实现“39证合一”，市本级和章贡区政务服务事项实现相对集中办理，“最多跑一次”事项占比超过80%，清理取消证明事项126项。原14家市属国有企业整合重组为七大集团。市县机构改革有序推进。农村集体资产清产核资全面完成，房地一体确权登记发证全面启动。科技创新水平有较大提升。R＆D【9】经费支出占GDP比重达1.2%，专利授权量、万人发明专利拥有量位居全省前列，科睿特成为全省唯一入选工信部制造业“双创”试点示范项目，稀土功能材料研究院成功创建省级制造业创新中心，中国联通（江西）工业互联网研究院在赣州成立运行。成功举办首届赣南苏区人才峰会，76人次的院士亲临指导，21位院士在赣州建立工作站，建成高层次人才产业园2个，引进国家“千人计划”专家27人、博士等高层次人才327人，赣州正成为高端人才创新创业的热土！“北上南下”成效显著。争取上级各类补助资金突破600亿元。赣南苏区振兴发展（北京）产业合作推介会、赣州（香港）经贸合作交流会等重大招商活动成果丰硕，签约项目183个，引进投资50亿元以上项目12个、超百亿元项目8个。赣州综合保税区开通至香港货运直通车，龙南保税物流中心、定南铁路集装箱码头建成运营，瑞金陆路口岸基本建成，全方位开放格局加速形成。

　　三是城乡面貌发生新变化。中心城区强心提质。东江源大道北延、南康苏访贤大道、赣县城北大道等城市道路建成通车，主次干道及支路“白改黑”基本完成，百姓盼望已久的105国道中心城区改线工程开工建设，赣州西客站建设进展顺利，市综合文化艺术中心开工建设，现代会展中心、全民健身中心前期工作全面完成。提升改造背街小巷1143条，拆墙透绿31.8公里，拆除“两违”建筑668万平方米、铁皮棚755万平方米，新建绿色建筑总量居全省前列。国家卫生城市创建顺利通过省级复审。农村人居环境加快改善。完成5516个新农村建设村点整治，农村生活垃圾治理通过国家验收，农村危旧“空心房”整治基本完成，超高超大建房基本得到遏制。实施国省道改造928公里，建设农村公路3945公里，建成“四好农村路”【10】2500公里，在全省率先实现组组通水泥路。赣州城市更具现代化都市气息，乡村更加整洁美丽、和谐宜居！

　　四是社会事业取得新进步。在财政收支压力加大的情况下，全市民生支出达725.1亿元，占一般公共预算支出的84.6%。省市90件民生实事完成年度计划。新开工学校139所，投入使用53所，新增学位7.76万个，学校建设力度、规模为历年之最。提前两年全域通过国家义务教育基本均衡发展评估认定，高考600分以上考生占全省比重提高6.1个百分点，一本上线万人比提高1.9。公立医院综合改革走在前列，在全省率先开展“先诊疗后付费”试点、率先启动公立医院取消医用耗材加成，组建运行2个市级医联体【11】、7个市级专科联盟【12】，赣南医学院一附院黄金院区全面投入使用，新建成乡（镇）卫生院35所、新增床位4000张，1351所村卫生计生服务室标准化建设基本完成。中心城区建成智能化停车场8个、新增停车泊位2100个，建成小游园18个、农贸市场18个，新（改、扩）建公厕157座。城市棚户区改造新开工4.69万套，基本建成5万套。在全国率先为城镇贫困群众缴纳居民养老保险。章贡区被列为全国智慧健康养老示范基地。《客家儿郎》登上全国舞台，《毛泽东寻乌调查》入选国家百部重点电视剧，电影《八子》顺利开拍。十五届省运会成绩名列前茅。安全生产形势稳中向好，各类事故起数、死亡人数大幅下降。扫黑除恶专项斗争形成强大震慑，刑事案件发案率明显下降，公众安全感和满意度全省第二，社会大局和谐稳定。国防教育、拥军优属、人民防空、应急管理、消防、审计、统计、民宗、外侨、对台、档案、地方志、老龄、残疾人、妇女儿童、红十字会等各方面工作取得新成效。

　　五是政府自身建设得到新加强。深入推进“两学一做”学习教育常态化制度化，严格落实中央八项规定及其实施细则精神，全面落实国务院“约法三章”。认真落实市委改作风、提效率“一个意见、两个办法”【13】“优化发展环境50条”“激励广大干部新时代新担当新作为的实施意见”，强力推进“五型”政府【14】建设，积极开展大众评公务和营商环境评价工作，深化扶贫领域腐败和作风问题专项治理，大力整治“怕、慢、假、庸、散”等作风顽疾，行政效能不断提升。法治政府建设成效明显，获评法治江西建设优秀单位，《客家围屋保护条例》《城市道路车辆通行管理规定》颁布实施。主动接受人大监督和政协民主监督，建议和提案办结率、满意和基本满意率均达100%。认真落实党风廉政建设和意识形态工作主体责任，坚决全面彻底肃清苏荣案余毒，全市政府系统党风廉政建设不断向纵深发展。

　　各位代表！过去一年，全市上下攻坚克难、担当实干，创造了多项全省有地位、全国有影响的赣州经验，有21个全国全省经验交流现场会在赣州召开，在35个全国全省会议上作典型发言；“财园信贷通、小微信贷通、创业信贷通”、会昌推广PPP模式【15】投融资体制改革、大余土地节约集约利用、于都公立医院综合改革等4项工作获国务院通报表彰；赣州被评为全国质量魅力城市、全省旅游产业发展先进市，安远被命名为“四好农村路” 全国示范县，崇义被评为全省十佳营商环境县，章贡区、会昌、龙南获全省发展非公有制经济先进县，全南获全国信访“三无县”，特别是我市连续两年获全省科学发展综合考评第一名。在各项任务繁重交织、困难压力叠加增大的情况下，能够取得这样的成绩，十分来之不易！这是党中央、国务院倾情关怀和国家部委鼎力支持的结果，是省委、省政府和市委坚强领导的结果，是全市人民团结一心、奋勇拼搏、纵深推进六大攻坚战的结果。在此，我代表市人民政府，向全市人民，向各民主党派、人民团体和社会各界人士，向驻市人民解放军、武警部队官兵，向中央、省属驻市单位，向中央对口支援单位及全体挂职干部，向所有关心支持赣州发展的海内外朋友，表示崇高的敬意和衷心的感谢！

　　需要向代表说明的是，受经济下行及投资、市场需求增速放缓等因素影响，固定资产投资、社会消费品零售总额增幅虽然高于全省0.2和0.1个百分点，但低于预期目标1.7和1.4个百分点。同时，我们也清醒看到，我市底子薄、基础弱，发展不平衡、不充分的问题仍然比较突出。自主创新能力不强，高质量发展任重道远；脱贫攻坚任务艰巨，民生和基础设施领域还有不少短板；风险防范、生态环保、安全生产等方面存在不少薄弱环节；一些政府部门及工作人员担当不够、服务意识不强、行政效率不高，发展环境仍需进一步优化，等等。对此，我们将采取有力措施，切实加以解决。

　　二、2019年工作安排

　　2019年是新中国成立70周年，是全面建成小康社会的关键之年。做好今年政府工作至关重要。令人振奋的是，在党中央、国务院的深切关怀下，国家发改委等部委于2月11日联合出台了支持赣南苏区振兴发展的若干政策措施，这是继《若干意见》【16】后国家层面为我市量身定制的又一重大政策文件，为赣南苏区振兴发展注入了新的强大动力。我们将感恩奋进、主动作为，奋力开创赣南苏区振兴发展新局面。

　　今年政府工作的总体要求是：坚持以习近平新时代中国特色社会主义思想为指导，深入贯彻党的十九大、十九届二中、三中全会和中央经济工作会议、省委十四届六次、七次全会和市委五届六次全会精神，从更高层次贯彻落实习近平总书记对江西和赣州工作的重要要求，坚持稳中求进工作总基调，坚持新发展理念，坚持高质量跨越式发展首要战略，坚持以供给侧结构性改革为主线，坚持深化市场化改革、扩大高水平开放，把中央打好三大攻坚战的部署要求贯穿经济社会发展全过程、各方面，统筹抓好稳增长、促改革、调结构、惠民生、防风险各项工作，进一步稳就业、稳金融、稳外贸、稳外资、稳投资、稳预期，坚定不移打好我市六大攻坚战，进一步抓好“两城两谷一带”建设，加快建设省域副中心城市，着力打造对接融入“一带一路”、粤港澳大湾区建设的桥头堡，深入推进“五型”政府建设，纵深推进赣南苏区振兴发展，奋力迈出打造新时代中国特色社会主义红色样板的坚实步伐，为全面建成小康社会收官打下决定性基础，以优异成绩庆祝中华人民共和国成立70周年。

　　今年发展主要预期目标是：生产总值增长9%左右，财政总收入增长7%左右，一般公共预算收入增长5%左右，规模以上工业增加值增长9%左右，固定资产投资增长10%左右，社会消费品零售总额增长11%左右，实际利用省外2000万元以上资金增长10%，实际利用外资增长7%左右，外贸自营出口增长5%左右，城镇居民人均可支配收入增长8.3%左右，农村居民人均可支配收入增长11%左右，居民消费价格总水平涨幅控制在3%左右，城镇登记失业率控制在4.5%以内，节能减排全面完成省下达任务。

　　围绕实现上述目标，重点抓好九个方面工作。

　　（一）坚决打赢精准脱贫攻坚战。向区域性整体脱贫发起总攻，确保南康、寻乌、安远、石城、上犹、会昌脱贫摘帽，赣县、兴国、于都、宁都达到脱贫摘帽要求，非贫困县完成减贫任务，贫困村全部脱贫退出，减少贫困人口15.69万人，贫困发生率降至0.42%左右。

　　确保两不愁三保障全面达标。全面解决贫困人口饮水安全、老人住危房等问题，完善健康扶贫“四道医疗保障线”【17】制度，不让一个孩子因贫失学辍学。聚焦140个深度贫困村，在新增资金、项目、政策、服务上予以倾斜，全力攻克坚中之坚。

　　全面提升脱贫攻坚质量。坚决完成中央和省委脱贫攻坚专项巡视反馈问题整改。对有发展条件和意愿的贫困家庭，确保有可持续增收的产业覆盖，或至少有1名贫困劳动力稳定就业。深入推进“百企帮百村”精准扶贫行动。因村施策推进贫困村人居环境整治，全面完成水电路网等基础设施建设任务。深化扶贫领域腐败和作风问题专项整治，重拳整治形式主义、官僚主义和腐败问题。

　　完善稳定脱贫长效机制。加大志智双扶力度，拔除贫困群众思想“穷根”。加强感恩教育，提高和稳定群众满意度。制定和落实脱贫摘帽后续帮扶措施，切实减少和防止贫困人口返贫。统筹推进城镇贫困群众脱贫解困工作，确保全面小康路上一个不少！

　　（二）乘势推进“主攻工业、三年再翻番”。制定“主攻工业、三年再翻番”实施意见，用三年时间，实现各地首位产业规上工业企业主营业务收入和高新技术企业数量、全市规上工业企业主营业务收入和工业固定资产投资再翻番，构建具有赣州特色的现代工业体系。

　　紧紧咬住集群发展。加快“两城两谷一带”建设。“新能源汽车科技城”重点加快国机、凯马整车下线投产，推进中电汽车、昶洧一期等项目竣工，加快建设孚能科技三期，全面启动核心零部件产业园建设。“现代家居城”重点推动南康家具提品质、强品牌，建设标准化、智能化车间，加快企业“升规入园”，扶持汇明木业上市，力争家具产业集群产值突破1800亿元。“中国稀金谷”重点推动重大项目落户核心区，做大做强南方稀土集团，建成稀金新材料研究院，持续向深加工及应用后端发力，有色金属产业产值达1000亿元以上、新材料及应用占比超过40%。“青峰药谷”重点推动青峰药业二期整体竣工及新上品种，加快高层次人才产业园项目孵化，引进更多大健康产业项目。“赣粤电子信息产业带”重点在建链、补链、强链上下功夫，加快众恒科技园等项目建设，新引进一批龙头企业、品牌厂商和整机制造企业，力争规上企业主营业务收入突破700亿元。培育壮大于都服装服饰、会昌氟盐化工等19个首位产业，打造沿东西方向的纺织服装产业带，用三年时间，新培育装备制造、电子信息、纺织服装等3个过千亿产业集群。

　　促进园区实力大提升。扎实开展“大干项目年”活动，国家级、省级开发区分别至少引进一个投资超50亿元、超20亿元的产业项目。全力推动园区做大做强、争先进位，力争全市工业园区主营业务收入突破3000亿元。强力推进工业用地“亩均论英雄”改革，加快处置“僵尸企业”，确保亩均投资强度、亩产效益分别提高到400万元、260万元以上。实施“两型三化”管理提标提档行动【18】，创建智慧开发区，年内所有开发区（园区）智慧平台与规模以上企业互联互通，实现园区事园区内办结。新建标准厂房300万平方米以上，力争三年再建1000万平方米。

　　助力企业快速成长。实施龙头企业培育计划，培育主营业务收入超10亿元企业5至10家、超50亿元企业2至3家。实施中小微企业成长工程，新增规上工业企业200家以上、“专精特新”企业40家、“小巨人”企业【19】5家、“瞪羚”企业【20】2至3家、“独角兽”企业1至2家。加大企业上市“映山红行动”推进力度，力争新增境内外上市企业3家以上，新三板挂牌企业实现县域全覆盖。

　　（三）纵深推进省域副中心城市建设。力争中心城区建成区面积达190平方公里、城市人口达190万左右，全市常住人口城镇化率达51.7%以上，推动新型城镇化向更高水平迈进。

　　激发区域发展新活力。加快五区一体化发展，做大做强中心城区。实行城市规划统一管理、城建项目统筹推进，增强五区联动发展的协同性。推进城市路网互联互通，建成东江源大道、客家大道西延快速路，完成创业路高架、赣南大道快速路90%以上工程量，以及105国道中心城区改线工程路基施工，三年内各功能区之间形成不少于2条交通干道连接互通。高品质打造高铁新区，建成赣州西站站房一期及东广场、站前广场高架系统，以亮丽新姿迎接高铁通车！高标准规划建设蓉江新区，全面建成起步区骨干路网，加速打造现代化城市新区。完善赣州国际陆港集疏运体系，建设高效便捷的“智慧港”。坚持扬优成势、错位发展，壮大区域增长板块。深入推进瑞兴于“3+2”经济振兴试验区建设，加快建设瑞兴于快速交通走廊，大力发展红色旅游、现代轻纺、现代农业、食品加工等产业；加快“三南”园区一体化发展，推动共建产业园取得突破性进展；启动会寻安生态经济区建设，规划建设绿色循环产业园，构建会寻安生态长廊；研究推进上犹、崇义、大余幸福产业示范区建设，打造区域经济新的增长极。

　　提升城镇功能与品质。全面实施城市治脏、治乱、治堵、功能修补、生态修复、特色彰显、亮化美化、治理创新“八大行动”。推进城市道路清扫保洁市场化、机械化、专业化、标准化，中心城区道路机扫率达到85%以上。提升章贡区水东、水西组团整体风貌，全面完成中心城区背街小巷提升改造及拆墙透绿。加快老旧小区改造，稳妥推进既有多层住宅加装电梯。完善城市交通体系，全面完成城市地铁规划编制，建成南河大桥拓宽改造一期桥梁主体工程，实施三康庙转盘交叉口改造，打通10条城市支路，推进道路地名管理规范化、科学化和标准化建设；加快城市公交、出租一体化，开通五区之间“大站快线”【21】公交，统一五区公交一卡通应用；加强停车设施建设，中心城区建成公共停车场17个以上，新增停车泊位2600个以上。大力推进城市“双修”【22】，建成试运行上犹江引水工程一期暨龙华水厂、市生活垃圾焚烧发电厂二期等项目；基本建成市综合文化艺术中心，完成现代会展中心、全民健身中心体育场主体50%工程量；中心城区完成12个农贸市场改造提升、新增社区蔬菜便利店30家、新（改）建8个市民休闲公园和30个小游园，全市新（改、扩）建公厕1857所，全面完成中心城区“厕所革命”任务；新建海绵城市【23】5平方公里以上，城市新建区域管线全部入廊入地。积极开展城市设计，启动实施快速路两侧建筑立面整治改造，大力实施绿化彩化及夜景照明提升工程，加强历史文化街区和传统风貌街区修缮保护。加快建设新型智慧城市，抓好中心城区“保文创卫”，推动县（市）积极创建文明、卫生、森林和园林城市。加强小城镇和特色小镇建设，非贫困县和已脱贫摘帽县每年至少启动建设1个新型城镇化示范镇。

　　实现基础设施新跨越。确保昌赣高铁建成通车，加快建设赣深高铁，力争开工长赣高铁，尽快形成“十”字型高铁枢纽。加快建设兴泉铁路，做好瑞梅铁路开工准备，加快赣郴永兴、赣韶扩能、南丰至瑞金城际铁路前期工作，启动赣广、赣龙厦高铁规划研究。完成兴赣高速北延项目50%工程量，开工建设大广高速南康至龙南段扩容工程、寻乌至龙川高速公路，启动信丰至南雄、井冈山至大余、赣州南至安远版石、寻全高速西延、济广与厦蓉高速会昌联络线等高速公路前期工作。完成黄金机场T2航站楼转场运营，实施T1航站楼一期改造，开工建设通用机坪，力争开通航空口岸和国际航线。全面开工建设瑞金机场，推动大余新城机场列为军民合用机场，争取江西飞行学院落户赣州，推进龙南、宁都、崇义、安远、兴国、信丰通用机场前期工作。开工建设赣州综合货运码头，开展赣粤运河前期研究，着力打通水运大通道。加快华能瑞金电厂二期机组主体建设，开工建设信丰电厂，建成赣州西500千伏等输变电工程，推进新一轮农村电网升级改造。推动县级应急备用水源全部开工建设。加快省天然气管网赣州段建设进度，力争12条支线全部具备通气条件，确保大余-信丰、瑞金-会昌、信丰-龙南、龙南-定南和于都支线、兴国支线等6条支线如期建成通气。

　　（四）开创乡村振兴新局面。深化农业供给侧结构性改革，加快发展现代农业，稳住“三农”这个基本盘。

　　扬优成势壮大现代农业。严守耕地红线，完成30.56万亩高标准农田建设。适度扩大脐橙种植规模，病毁果园复种和新开发果园7万亩以上，新建标准化生态果园150个。推进蔬菜全产业链发展，新增钢架大棚8万亩以上，规模蔬菜基地突破30万亩，建立技术服务和市场营销体系，打响“赣南蔬菜”品牌。抓好油茶高产稳产示范基地建设，新造高产油茶林8万亩，改造低产油茶林5万亩，引导规范使用“赣南茶油”品牌商标。每个县（市、区）集中发展1至2个农业特色产业，推动黄鸡、麻鸭、灰鹅、肉牛、茶叶、白莲、刺葡萄等产业规模化、品牌化发展。推进生猪产业转型升级，积极发展林下经济。加强柑橘黄龙病、非洲猪瘟、松材线虫病等重大动植物疫病防控。全面清理整治“大棚房”【24】，坚决遏制农地非农化。深入实施农产品加工业提升行动，大力培育和引进畜禽屠宰加工和果蔬类龙头企业。积极发展农产品物流、电商等新业态，加快农村一二三产业融合发展，让全市农民获得更多产业链的增值收益和就业岗位。

　　推动农业农村改革走深走实。完善农村承包地“三权分置”【25】办法，将农村土地流转率提高到40%以上。深化农村集体产权制度改革，再扩大1000个村组试点集体资产股份权能改革。启动农村宅基地改革试点。多途径发展壮大集体经济，力争每个行政村村级集体经济年均经营性收入过5万元。推进农业经营体制改革，培育发展产业联盟、农业产业化联合体，新增农民合作社800家、家庭农场500个，把小农户引入现代农业发展大格局。

　　建设生态宜居美丽乡村。深入推进农村人居环境整治三年行动计划。因地制宜做好村庄规划，加强农村建房管理，持续整治超高超大建房，有效管控农村风貌。完成5000个左右新农村建设村点整治，今年预退出的4个贫困县（区）实现25户以上自然村组整治全覆盖。推行农村环卫“全域一体化”第三方治理和保洁员全职化，具备条件的村庄完成生活污水处理设施建设，完成农户改厕2.1万户。实施国省道改造880公里，建设农村公路1750公里，基本建成“四好农村路”。持续深入开展乡风文明行动，积极稳妥推进绿色殡葬改革，破除陈规陋习，培育新时代文明乡风。

　　（五）繁荣壮大现代服务业。优化商贸供给质量，激活赣州消费大市场，力争服务业增加值突破1500亿元。

　　做旺文化旅游。强力推动“一核三区”【26】建成一批核心景区，力争完成旅游项目投资300亿元，旅游综合收入达到1300亿元。加快文化强市建设步伐，促进商旅文深度融合、一体化发展。打响“红色故都”品牌，建成红色文化展示馆（中央苏区历史博物馆），加快复兴之路主题公园、长征国家文化公园（于都段）、瑞金红色演艺等项目建设，深化与长征沿线城市交流合作，加快形成以瑞金为龙头，兴国、于都为重点，宁都、石城、寻乌、会昌、安远、大余、信丰等地多点支撑的红色旅游协同发展格局，将赣州打造成全国红色旅游“一线城市”。打响“江南宋城”品牌，高标准建设宋城历史文化街区，整合水西旅游板块，开发水上观光、实景演出、主题娱乐等旅游产品，打造人气旺盛的“三江六岸”旅游区。打响“客家摇篮”品牌，加快龙南世界围屋博览园等项目建设，推动安远三百山创建国家5A级景区，活化利用赣南围屋和客家文化资源，筹办客家文化旅游节、世界客家围屋高峰论坛等活动，打造国内知名的客家文化体验旅游区。打响“阳明文化”品牌，加快阳明湖、天沐温泉等景区建设，形成“阳明文化+绿色生态”的特色旅游产品。完善旅游要素配套，加快建设星级酒店、星级旅行社、游客集散中心、旅游厕所，力争开业运营5家五星级、15家四星级酒店，每个县（市、区）建成1家旅游商品旗舰店、1家客家美食旗舰店，推出一桌特色菜，打造具有赣南特色的名宴、名菜、名点、名小吃等客家美食名片。

　　做活现代金融。积极创建普惠金融改革试验区。启动建设蓉江新区金融商务核心区。深入推进区块链金融产业沙盒园【27】建设。争取瑞京人寿获批。完成稀有金属交易所重组。做大做强地方法人机构，支持赣州银行引进战略投资者。推进“险资入赣”，扩大债券融资规模，力争企业直接融资超200亿元，新增信贷700亿元以上，让更多金融“活水”浇灌实体经济。

　　做大商贸物流。加快建设高铁站前商务中心，建成运营杉杉奥特莱斯，新建大型商贸综合体，打造高品位特色商业街区，形成城市核心商圈。推进赣州综合物流园二期、赣州智慧供应链产业园等22个重点物流项目建设，建成投用赣州冷链物流中心。建设农产品批发市场等商品交易市场，构建城乡高效物流配送网络体系。积极培育发展无人机快递、智慧物流、物流金融等新业态，打造赣州物流大数据中心。

　　做特康养品牌。深入实施健康养老产业发展三年行动，打造一批健康养老示范综合体。推进居家和社区养老服务改革试点，新增居家养老服务站点200家以上，每个县（市、区）培育打造2至3家专业化养老服务社会组织，加快构建具有赣南特色的养老体系。充分利用生态优势、温泉资源和旅游景区，推动“康养+旅游”融合发展，打造全国知名的康养旅游目的地。

　　（六）构建更高层次改革开放新格局。深化市场化改革，扩大高水平开放，为高质量跨越式发展注入强劲动力。

　　全力打造“四最”【28】营商环境。推动“放管服”改革取得革命性突破。与企业和群众日常生产生活密切相关的办事窗口实行“全天候”服务。全面启用“12345”政务服务热线，上线运行“一证通办”系统和“赣州通”，实现高频服务事项“掌上办”。“最多跑一次”事项占比提高到90%以上，“一次不用跑”“就近办”事项提高到50%以上。全面推行“证照分离”【29】改革，实现企业开办全程“网上办、零见面”。推进投资项目审批提质增效，审批时间再压减20%以上。深化相对集中行政许可权改革试点，创新“一窗受理+一章审批+智慧政务”模式，市县两级依申请类政务服务事项网办率、“一窗式”受理率达70%以上。精准推进降成本优环境专项行动，全面落实“优化发展环境50条”。深入实施“双随机、一公开”【30】市场监管，对违法者“利剑高悬”、对守法者“无事不扰”。出台支持民营经济发展的政策措施，大力实施民营企业精准帮扶工程，帮助解决融资难、招工难等实际困难，助力民营企业跨越市场的“冰山”、融资的“高山”、转型的“火山”。

　　聚焦重点领域改革攻坚。全面完成机构改革任务，稳步推进事业单位改革。深化国资国企改革，推进市属平台公司市场化转型，鼓励开展市场化并购，提升核心竞争力。深化财税体制改革，全面实施预算绩效管理。加大防范处置非法集资力度，加强互联网金融风险专项整治，稳妥处置地方政府债务风险，做到坚定、可控、有序、适度。构建房地产市场平稳健康发展长效机制，稳地价、稳房价、稳预期。

　　构筑内陆地区双向开放高地。加大“北上”力度，用足用好《若干意见》，全面对接落实《若干措施》，全力争取国家启动制定2020年之后新一轮支持赣南苏区振兴发展的政策意见。突出招大引强，深耕珠三角、紧盯闽东南、拓展长三角，精准实施专业招商、中介招商、并购招商，大力开展“赣深经贸活动周”“三请三回”【31】“三企入赣”【32】等招商活动，推动“赣商回归”“赣才回归”，力争引进投资超10亿元项目50个以上、超50亿元项目10个以上、超百亿元项目5个以上。举全市之力做大做强赣州国际陆港，力争多式联运二期工程上半年全面建成，推动中欧（亚）班列增点扩线，建成运营肉类、汽车整车进口指定口岸，申报进境粮谷、药品等指定口岸，争取获批在安远建设进境水果口岸指定作业区；（下转3版）　　（上接2版）

　　启动建设跨境电商产业园，争取获批国家跨境电商综合试验区；推进赣州国际陆港与赣州综合保税区、航空口岸、高铁新区、新能源汽车科技城和龙南、瑞金、定南口岸作业区联动发展，推动实现货物“进境与沿海同价到港、出境与沿海同价起运、通关与沿海同等效率”。深化区域开放合作，推动与广州、深圳、厦门、宁波等地合作共建事项落地见效，着力把赣州打造成为产业梯度转移承接示范地、赣粤开放合作高地、对接融入粤港澳大湾区建设的桥头堡。

　　（七）深入实施创新驱动战略。以建设“中国制造2025”试点示范城市为引领，积极打造区域性科研创新中心、国家创新型城市。

　　大幅提升科技创新能力。加大全社会研发投入攻坚力度，R＆D经费支出占GDP比重提高至1.6%以上。实施科技型企业培育工程，新增高新技术企业100家以上。推动200家企业技改，完成技改投资200亿元以上。加快推进6个智能工厂、20个数字车间建设，新建“机器换人”项目30个以上。充分发挥高层次人才产业园作用，对接落实赣南苏区人才峰会成果，力争新引进产业领军人才20名以上、高层次人才200名以上，让赣南成为人才荟萃、实现梦想的家园！

　　加速创新成果落地转化。围绕产业链部署创新链，聚焦“两城两谷一带”实施重大关键技术攻关，积极承担省级重大科技专项，组建省级科技协同创新体。加快稀金科创城、启迪科技城建设，加强与国内知名院所高校协同创新，争取设立分支研究机构、产业研究院，整合组建一批省级以上重点实验室、企业技术中心，新增省级以上科技孵化器和众创空间8个以上，争取“中国稀金谷”列为国家双创示范基地。实施专利质量提升工程，力争万人发明专利拥有量提高到2件。

　　打响赣州数字经济品牌。出台数字产业发展政策，培育一批数字产业龙头骨干企业，建成蓉江新区赣南数据湖示范园，加快章贡区软件物联及创新创意孵化园建设，推动南康、赣县、赣州经开区等地发展家居、物流、稀有金属、金融等智慧应用产业。深化“03专项”【33】建设，推进4G网络深度覆盖，试点开展5G应用，为迈入5G时代奠定良好基础。

　　（八）全面加强生态文明建设。重拳出击、标本兼治，持续打好污染防治攻坚战，争当国家生态文明试验区建设排头兵。

　　铁腕整治环保突出问题。对标抓好中央和省环保督察反馈问题整改，确保环境质量保持全省前列。分类整治“散乱污”企业，完成污染较重企业搬迁改造年度计划。加快推进赣县红金工业园一期企业搬迁，完成瑞金万年青水泥公司卫生防护距离内住宅搬迁任务一半以上。如期完成违规小水电清理整顿、饮用水水源地环境问题整改计划，抓好乡镇集中式饮用水水源地保护区划定及规范化建设。加强重金属重点防控，全面推进钨渣处理处置。纵深推进蓝天保卫战，确保中心城区PM2.5浓度均值持续优于省考核目标值，各县（市）PM2.5浓度均值下降8%以上。巩固提升消灭劣Ⅴ类水成果，深入推进畜禽养殖污染整治“回头看”，确保断面水质稳定达标升级。

　　强化生态环境综合治理。推进山水林田湖草生态保护修复18个年度项目开工建设，加强废弃稀土矿山后期管护，完成低质低效林改造110万亩。加快生态环保基础设施建设，完成建筑垃圾资源化利用处理场主体工程，建成运行餐厨废弃物资源化利用处理中心以及信丰、瑞金生活垃圾焚烧发电厂，建成污水管网180公里以上，城镇生活污水处理率提高到93%以上。

　　创新完善生态文明制度。完善空间用途管制措施，严守生态保护红线、基本农田控制线、水资源保护红线。积极探索开展碳排放权、排污权、用能权、水权等交易试点。争取实施第二轮东江流域上下游横向生态补偿。完善生态文明建设考核评价、约谈问责制度，全面推行领导干部自然资源资产离任审计，以最严格的制度、最严密的法治守护好赣南的绿水青山！

　　（九）持续加力保障和改善民生。坚持以人民为中心的发展思想，高质量完成省下达51件和我市40件民生实事，不断增进民生福祉，让人民群众拥有更多获得感幸福感安全感！

　　办好人民满意教育。深入实施第三期学前教育行动计划，年内全市建设公办幼儿园160所以上，新增住宅小区配建普惠性民办幼儿园40所以上，加快建设县级儿童活动中心，抓好贫困地区儿童早期教育，促进学前教育普及、普惠、安全、优质发展。巩固义务教育基本均衡评估成果，全面完成555所薄弱学校改造，建设中小学校60所、建成投入使用25所，中心城区建成或基本建成3所普通高中，填补10年未建高中的空白。大力实施第二期特殊教育提升计划，新建赣州市特殊教育学校，加快推进各县（市、区）特殊教育学校及特殊教育资源中心建设。加快建设赣州职教园区，建成投用赣南职业技术学院。全面深化新时代教师队伍建设改革，实施教师能力提升工程，营造尊师重教良好氛围。

　　加快建设健康赣州。完成市妇保院新院主体工程一半以上，新（改、扩）建市中医院新院、市第三人民医院新院、赣州经开区人民医院、赣县区中医院、南康区第一、第二人民医院，推动南康、信丰、于都、宁都、瑞金、兴国创建三级综合医院，新（改、扩）建乡镇卫生院54所。深化医药卫生体制改革，把更多救命救急的好药纳入医保，在市级及以上医院全面推行掌上预约诊疗，设立重症患者医疗检查专区，探索名医跨区域灵活行医机制；完善分级诊疗制度，力争县域内就诊率达90%左右，推动县域内住院“先诊疗后付费”、“一站式”结算、扶贫病床设置和大病集中救治全覆盖。举办第五届市运会，加快建设“新时代重走长征路”健身步道，让人民群众享受运动快乐、拥有健康体魄。

　　兜牢民生保障底线。全面落实促进就业创业政策，重点解决高校毕业生、农民工、退役军人等群体就业，确保零就业家庭动态“清零”。落实全民参保计划，对未参保人员实施精准扩面，提高城乡居民基本养老、基本医保和低保等补助标准，稳步提升特困人员供养水平。深入实施保障性安居工程，完成城市棚户区改造1.6万户，全面解决返迁安置房建设历史遗留问题。大力发展社会救助、公益慈善、残疾康复等事业，切实做好托底工作，让每一个身处困境者得到关爱和温暖。

　　全力维护社会稳定。深化平安赣州、法治赣州建设。完善公共法律服务体系，推进农村“法律明白人”培养工程。加快基层综治中心实体化建设，大力推进“雪亮工程”【34】，完善立体化、信息化社会治安防控体系，创建全国社会心理服务体系建设试点城市。深入践行新时代“枫桥经验”【35】，创新基层社会治理，打造社会治理共同体，加强和改进信访维稳工作。持续推进民族团结进步，依法维护宗教和谐稳定。纵深推进扫黑除恶专项斗争，深挖彻查“保护伞”。深入创建国家食品安全示范城市，加强食品药品监督管理。强化应急管理体系建设，深入开展安全生产十大专项整治行动，坚决防范遏制重特大事故发生，切实保障人民群众生命财产安全。

　　扎实推进国防动员、国防教育、拥军优属、国家安全、人民防空、退役军人事务、消防、外侨、对台、气象、地质、老龄等工作，大力支持工、青、妇、红十字会等群团组织建设，让各行各业充满生机活力！

　　各位代表！使命呼唤担当，奋斗成就梦想。全市政府系统将不忘初心、牢记使命，持续推进“五型”政府建设，努力打造人民更加满意的政府。我们将着力打造忠诚型政府，树牢“四个意识”，坚定“四个自信”，坚决做到“两个维护”，坚决对标对表中央精神，坚决落实省委、省政府和市委决策部署，争当最讲党性、最讲政治、最讲忠诚的表率。着力打造创新型政府，认真开展大学习、大调研，开拓新视野、研究新情况、适应新要求，推动思想大解放、能力大提升、职能大转变、工作大创新，争创新时代“第一等工作”。着力打造担当型政府，从严整治形式主义、官僚主义等作风顽疾，大力弘扬苏区精神和苏区干部好作风，让“不为不办找理由、只为办好想办法”成为各级政府及其工作人员的鲜明标识，让“事事马上办、人人钉钉子、个个敢担当”在政府系统蔚然成风。着力打造服务型政府，坚决向不作为、慢作为、乱作为“亮剑”，全面推行“店小二”“保姆式”服务，充分运用大数据、云计算、物联网等新技术，大幅提升政务服务效率，最大限度便民利企，真正让企业和群众办事少跑腿、不闹心！着力打造过硬型政府，推动政府系统全面从严治党向纵深发展，严格落实中央八项规定及其实施细则精神，认真履行党风廉政建设和意识形态工作责任制，全面推进法治政府建设，自觉接受人大及其常委会法律监督和工作监督、政协民主监督以及监察、司法、社会、舆论等各方面监督，持之以恒正风肃纪，全力巩固风清气正的政治生态！

　　各位代表！全面振兴的光明前景令人振奋，全面小康的进军号角催人奋进。让我们更加紧密地团结在以习近平同志为核心的党中央周围，在省委、省政府和市委的坚强领导下，担当实干，奋发有为，加快推进赣南苏区振兴发展，奋力打造新时代中国特色社会主义红色样板，以优异成绩庆祝中华人民共和国成立70周年！
